# “Hang Ups, Let Downs, Bad Breaks, Setbacks”: Impact of Structural Socioeconomic Racism and Resilience on Cognitive Change Over Time for Persons Racialized as Black

**DOI:** 10.1089/heq.2023.0151

**Published:** 2024-04-15

**Authors:** Paris B. Adkins-Jackson, Boeun Kim, César Higgins Tejera, Tiffany N. Ford, Ariana N. Gobaud, Kyler J. Sherman-Wilkins, Indira C. Turney, Justina F. Avila-Rieger, Kendra D. Sims, Safiyyah M. Okoye, Daniel W. Belsky, Tanisha G. Hill-Jarrett, Laura Samuel, Gabriella Solomon, Jack H. Cleeve, Gilbert Gee, Roland J. Thorpe, Deidra C. Crews, Rachel R. Hardeman, Zinzi D. Bailey, Sarah L. Szanton, Jennifer J. Manly

**Affiliations:** ^1^ Department of Epidemiology, Mailman School of Public Health, Columbia University, New York, New York, USA.; ^2^ Department of Sociomedical Sciences, Mailman School of Public Health, Columbia University, New York, New York, USA.; ^3^ School of Nursing, Johns Hopkins University, Baltimore, Maryland, USA.; ^4^ Department of Epidemiology, University of Michigan School of Public Health, Ann Arbor, Michigan, USA.; ^5^ Division of Community Health Sciences, School of Public Health, University of Illinois Chicago, Chicago, Illinois, USA.; ^6^ The Brookings Institution, Washington, District of Columbia, USA.; ^7^ Department of Sociology and Anthropology, Missouri State University, Springfield, Missouri, USA.; ^8^ Department of Neurology, Vagelos College of Physicians & Surgeons, Taub Institute for Research on Alzheimer's Disease & The Aging Brain, Columbia University, New York, New York, USA.; ^9^ Department of Epidemiology and Biostatistics, University of California, San Francisco, San Francisco, California, USA.; ^10^ Department of Graduate Nursing, College of Nursing and Health Professions and Dornsife School of Public Health, Drexel University, Philadelphia, Pennsylvania, USA.; ^11^ Department of Health Management and Policy, College of Nursing and Health Professions and Dornsife School of Public Health, Drexel University, Philadelphia, Pennsylvania, USA.; ^12^ Butler Columbia Aging Center, Mailman School of Public Health, Columbia University, New York, New York, USA.; ^13^ Department of Neurology, Memory and Aging Center, University of California San Francisco, San Francisco, California, USA.; ^14^ Department of Community Health Sciences, University of California at Los Angeles, Los Angeles, California, USA.; ^15^ Alzheimer's Disease Resource Center for Minority Aging Research, Johns Hopkins University, Baltimore, Maryland, USA.; ^16^ School of Medicine, Johns Hopkins University, Baltimore, Maryland, USA.; ^17^ Center for Antiracism Research for Health Equity, University of Minnesota School of Public Health, Minneapolis, Minnesota, USA.; ^18^ Department of Medicine, University of Miami Miller School of Medicine, Miami, Florida, USA.; ^19^ Department of Public Health Sciences, University of Miami Miller School of Medicine, Miami, Florida, USA.

**Keywords:** structural racism, socioeconomic status, cognition

## Abstract

**Introduction:**

Older adults racialized as Black experience higher rates of dementia than those racialized as White. Structural racism produces socioeconomic challenges, described by artist Marvin Gaye as “hang ups, let downs, bad breaks, setbacks” that likely contribute to dementia disparities. Robust dementia literature suggests socioeconomic factors may also be key resiliencies.

**Methods:**

We linked state-level data reflecting the racialized landscape of economic opportunity across the 20th Century from the U.S. Census (1930–2010) with individual-level data on cognitive outcomes from the U.S. Health and Retirement Study participants racialized as Black. A purposive sample of participants born after the Brown v. Board ruling (born 1954–59) were selected who completed the modified Telephone Interview for Cognitive Status between 2010 and 2020 (*N*=1381). We tested associations of exposure to structural racism and resilience before birth, and during childhood, young-adulthood, and midlife with cognitive trajectories in mid-late life using mixed-effects regression models.

**Results:**

Older adults born in places with higher state-level structural socioeconomic racism experienced a more rapid cognitive decline in later life compared to those with lower levels of exposure. In addition, participants born in places with higher levels of state-level structural socioeconomic resilience experienced slower cognitive change over time than their counterparts.

**Discussion:**

These findings reveal the impact of racist U.S. policies enacted in the past that influence cognitive health over time and dementia risk later in life.

## Introduction

In 2022, over 10% of the United States population aged 65 or older (6.5 million) lived with dementia.^1–3^ However, the disease burden is unequal; older adults racialized as Black experience 1.5–1.9 times higher incidence compared with older adults racialized as White^4–6^ and suffer steeper cognitive decline.^7^ These profound Black-White disparities in cognitive health stem from lifetime exposure to structural racism, a fundamental cause of health disparities.^8^

Structural racism is the totality of ways that society unequally distributes resources across racialized groups.^9–11^ Across U.S. history, resources were distributed in ways yielding large disparities in socioeconomic opportunities between groups racialized as Black and White.^12–14^ Marvin Gaye, an artist racialized as Black, encapsulated the socioeconomic impact of racism with the refrain: “hang ups, let downs, bad breaks, setbacks. Natural fact is, honey, that I can't pay my taxes. Make me want to holler…”^15^ Gaye's 1971 album *What's Going On* illustrated how prevailing socioeconomic conditions influenced the daily wellbeing of marginalized groups.^15^ Literature examining socioeconomic resources has documented associations of low levels of education, income, and occupational prestige and complexity with cognitive decline and the onset of dementia.^6,13–24^

Research on education reveals the starkest racial disparities in dementia and cognitive decline. There is also a negative association between income and risk of dementia,^25^ and emerging research shows an association between occupational complexity and prestige with cognitive decline.^26,27^

Parallel literature suggests that these associations reflect the contribution of socioeconomic opportunities and resources to the development of cognitive reserve, the brain's ability to maintain cognitive functioning in the presence of neuropathology and neurodegeneration.^28,29^ Stark Black-White disparities in socioeconomic opportunities and resources explain Black-White disparities in cognitive aging. Socioeconomic opportunities and resources have also been strategically obtained by minoritized groups, like those racialized as Black, to reduce the burden of structural racism. From the development of economic metropolises like Black Wall Street in Oklahoma and North Carolina, to the creation of Historically Black Colleges and Universities, to the desegregation of neighborhoods and jobs, people racialized as Black have always combated structural racism through socioeconomic gains.

Despite the agency of people racialized as Black to create better socioeconomic opportunities and resources, little is known about how socioeconomic opportunities as mechanisms of structural resilience (structural determinants that interlock and reinforce each other toward health promotion)^30^ contribute to cognitive reserve in adults racialized as Black.^31^

This study explores the relationship between life course exposure to structural socioeconomic racism and structural socioeconomic resilience with cognitive change over time in a national sample of older adults racialized as Black (age ≥51). We constructed a sample of persons racialized as Black (born 1954–1959) who entered a post Brown v. Board of Education school system working toward desegregation and who endured a segregated workforce in the 1980s^32–35^ where high school diplomas became prerequisites for certain jobs. This sample experienced restricted access to cognitively engaging work that paid a living wage.^36,37^

Moreover, when this sample's cohort accumulated wealth, as with their parents, they were met with *de jure* residential segregation,^38,39^ the residual effects of *de facto* policies on impoverishment^40,41^ and a lack of economic mobility that stifled their neighborhoods.^42–46^ This study hypothesized that greater life course structural socioeconomic racism is associated with faster cognitive decline, and greater life course structural socioeconomic resilience is associated with slower cognitive decline in older adults racialized as Black.

## Methods

This retrospective cohort study linked historical U.S. Census state-level structural socioeconomic racism and resilience data to cognitive decline data with older adults racialized as Black in the Health and Retirement Study (HRS). Columbia University Institutional Review Board approved the study.

### Sample

The HRS is an ongoing longitudinal survey of a nationally representative sample of U.S. adults aged 51 years or older.^47^ The initial cohort began in 1992, and since 1998, younger cohorts (ages 51–56) are added every 6 years.^48^ For this study, we included persons racialized as Black born between 1954 and 1959. Cognitive test performance was collected biannually. HRS recorded the state where participants lived at birth, age 10, and interview.

### Study variables

#### Cognitive performance

The Telephone Interview for Cognitive Status (TICS) captures multiple domains of cognitive function, including an immediate and delayed 10-item word recall test, a serial 7 subtraction test, and a backwards count test starting from 20.^49^ Total TICS scores ranged from 0 to 27, with a higher score indicating better cognitive performance.^50^ We used cognitive performance data between 2010 and 2020 for both telephone and in-person and used imputed data provided for missing scores at baseline (4.8%).^51^

#### Life course structural socioeconomic racism and resilience

**FIG. 1.**
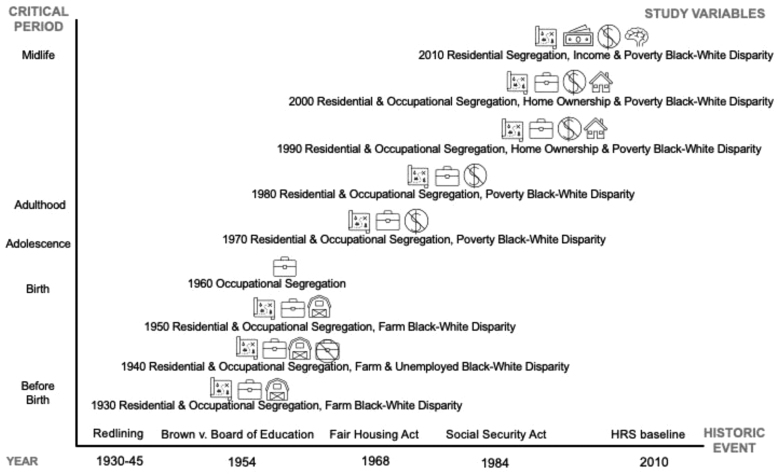
Life course structural socioeconomic racism by critical period, year, and historic event for Baby Boomers racialized as Black born 1954–1959.

The authors met regularly in 2022 to identify relevant socioeconomic mechanisms of structural racism and structural resilience. These variables capture population-based Black-White disparities that result from processes of racialization and discrimination that occurred between 1930 and 2010.^52^ The structural racism measure consists of items (*K*=31, Fig. 1) that capture decennial residential (1930–2010) and occupational segregation (1930–2000; 1950 occupational classification system used), disparities in income and poverty (1969, 1979, 1989, 1999, and 2008–2010), farm land (1930–1950), and owner-occupied units (1990–2000) obtained from Census via IPUMS (see configuration in Data Analysis section). The structural resilience measure uses of the same data to capture Black socioeconomic resources in the same timeframe.^53,54^

#### Covariates

To isolate the effects of structural factors from individual-level characteristics, we included covariates for participant-level education in our models^1,6^ (high school, high school graduate, some college), living arrangement (married/partnered living with partner, married/partnered not living with partner, living with others, living alone), cisgender (female/male), and age.

### Data analysis

For ease with replication and interpretation by interdisciplinary scholars, we used the index of concentration at the extremes (ICE)^55–57^ to standardize the structural socioeconomic racism measure and generated scores for each item per county from 1930 to 2010. ICE is the difference in the number of people racialized as White and Black for each category, divided by the total population, which changed over time based on data availability. In the denominator, for 1930–1960, persons racialized as White and non-White were included; 1970–1990, persons racialized as White, Black, American Indian and Alaskan Native, Asian and Pacific Islander, and Other were included; 2000–2010 additionally included persons racialized as Multiracial.

To avoid duplication due to the way the U.S. Census separates “race” from “ethnicity,” we excluded persons who were grouped as “Hispanic.” ICE scores range from −1 to 1, where −1 is the extreme concentration of persons racialized as Black and 1 is the extreme concentration of persons racialized as White.

After computing county-level ICE from 1930 to 2010, we averaged the values to obtain state-level ICE (*N*=52, including the District of Columbia and Puerto Rico). Because segregation can occur on both ends of the −1 to 1 interval, we used the absolute value for ICE, where 0 indicates integration, and 1 indicates extreme concentration of persons racialized as Black or White, suggesting extreme structural racism. Given discourse that structural racism is a latent variable,^58,59^ we performed a series of confirmatory factor analyses (CFA) with the averaged state-level absolute ICE with multidimensional (employment, income, other factors)^9,60^ and unidimensional (“interlock”) models.^61–63^

CFA model fit parameters included item loadings (>0.5), root mean square error of approximation (<0.06), comparative fit index (0.95), Tucker–Lewis index (>0.90), and standardized root mean square residual (<0.08). We entered all state-level items into a principal axis factor analysis (i.e., exploratory factor analysis [EFA]) with an Oblimin rotation for items to freely load to observe the dimensionality of structural socioeconomic racism. In the EFA, we also examined item loadings (>0.3) and communalities (>0.5), as well as the Kaiser-Meyer-Olkin (KMO) measure of sampling adequacy (>0.8) and Barlett's test of sphericity chi-square (*p*<0.05).^64,65^ Socioeconomic racism factor scores were computed for before birth, childhood/adolescence, adulthood, and midlife periods.

Based on the hypothesis that greater socioeconomic outcomes in a region reflect resilience, we used similar items for the structural socioeconomic resilience measure with different computations. We standardized the measure of items by computing the proportion Black score for U.S. counties where the number of people racialized as Black was divided by the total population for each item between 1930 and 2010. For years 1930–1960, the numerator was persons racialized as non-White; for all other years, the numerator included persons racialized as Black. The same denominator as ICE was used. Similarly, we averaged county percentage Black to the state-level per item, performed CFAs and an EFA with an Oblimin rotation, and computed socioeconomic resilience factor scores based on the same life course periods.

We used linear mixed-effects models with random intercepts and slopes to test the association between socioeconomic racism and resilience with cognitive change. We created a time metric based on the wave of the cognitive test scores (six waves). First, we ran models hierarchically for each main effect of exposure (e.g., before birth) with an interaction term between life course period and wave (e.g., before birth×wave) to test the association between socioeconomic racism and resilience by life course period and changes over time adjusting for covariates at baseline (two-sided *p*<0.05). We ran a full model, including all life course periods. Finally, we computed descriptive statistics of baseline characteristics. Data analyses were performed using IBM SPSS Statistics (Version 28), Mplus (Version 8.4), and Stata (Version 17).^66,67^

## Results

### Participant characteristics

A total of 1381 HRS participants were included in this analysis (Table 1). Most participants (86%) began the study in 2010 and fewer participants started prior 1992–2008 or after 2012–2020. Nearly 2% of participants also identified as Latine/x/a/o, 9% were born outside of the United States, 58% were cisgender female, 58% were married/partnered, 3% were living with potential dementia,^49^ and 55% had a high school diploma/General Educational Development at baseline. The mean global cognitive score was 14.0 (standard deviation [SD]=3.9) in 2010.

**Table 1.** Sample characteristics (*N*=1381)

Category	***N***	%
Born		
1954	197	14.3
1955	243	17.6
1956	223	16.2
1957	234	16.9
1958	244	17.7
1959	240	17.4
Entry wave		
Before 2010	96	7
2010	1192	86.3
After 2010	93	6.7
Total transitioned (died)		
Before 2014	39	2.8
After 2014	94	6.8
Latinx	23	1.7
Born outside the United States	119	8.6
Cisgender category		
Cisgender female	798	57.8
Cisgender male	583	42.2
Highest degree obtained		
Less than high school	237	17.2
High school degree	765	55.4
Some college and above	379	27.7
Living arrangement		
Married or partnered, living with partner	645	46.7
Married or partnered, not living with partner	106	7.7
Living with other	299	21.7
Living alone	250	18.1
Missing	81	5.9
Living with dementia (via Langa-Weir classification) in		
2010	39	2.8
2012	69	5
2014	97	7
2016	107	7.8
2018	106	7.7
2020	102	7.4

	M	SD
Before Birth (*N*=1086)		
Absolute ICE	−0.620	1.351
Percentage Black	0.740	1.364
Childhood/adolescence (*N*=1044)		
Absolute ICE	−0.665	0.806
Percentage Black		
Adulthood (*N*=1315)		
Absolute ICE	−0.505	0.879
Percentage Black	0.454	0.833
Midlife (*N*=1315)		
Absolute ICE	−0.494	0.867
Percentage Black	0.525	0.951
Global cognitive score in		
2010 (*N*=1263)	14.0	3.9
2012 (*N*=1181)	14.0	4.1
2014 (*N*=1120)	14.1	4.2
2016 (*N*=1047)	14.3	4.4
2018 (*N*=832)	14.1	4.2
2020 (*N*=807)	14.5	4.8

ICE, index of concentration at the extremes; SD, standard deviation.

### Measure of life course structural socioeconomic racism

The CFA for the multidimensional model could not converge to produce model fit. The unidimensional model did not meet model fit criteria. The EFA yielded a three-factor model accounting for roughly 78% of the total variance in structural socioeconomic racism explained by life course periods: Factor 1 (eigenvalue 13.63; 64.89% variance) comprised of items between 1970 and 2010, Factor 2 (eigenvalue 1.63; 7.75% variance) of items between 1930 and 1950, many of which cross-loaded with Factor 3 (eigenvalue 1.16; 5.53% variance) items relating to farm or home property value—all items had adequate communalities (Table 2). KMO measure of sampling adequacy and Barlett's test of sphericity chi-square were adequate.

**Table 2.** Exploratory factor analyses for structural socioeconomic items for both index of concentration at the extremes and percentage Black

Life coursepPeriod	Domain	Items	***N***	Mean ICE (SD)	Median ICE	Skewness (kurtosis)	ICE EFA factor loadings					Mean % Black (SD)	Median % Black	Skewness (kurtosis)	% Black EFA factor loadings				
							1930–1950	1960–1970	1980–2000	2010	Communalities				1930–1950	1960–1970	1980–2000	2010	Communalities
Before birth	Employment	Occupational segregation (1930)	51	0.92 (0.08)	0.95	−0.99 (−0.45)	0.817				0.667	0.05 (0.06)	0.030	1.22 (0.043)	0.837				0.701
		Occupational segregation (1940)	51	0.89 (0.19)	0.94	−3.67 (14.95)	0.468				0.219	0.06 (0.09)	0.030	3.52 (17.1)	0.664				0.441
		Occupational segregation (1950)	51	0.89 (0.17)	0.93	−3.59 (15.85)	0.486				0.236	0.07 (0.11)	0.030	4.09 (21.9)	0.592				0.351
		Black-White disparity in partly unemployed (1937)	51	0.78 (.32)	0.96	−1.31 (0.12)	0.886				0.785	0.12 (0.18)	0.020	1.49 (0.907)	0.845				0.714
	Income/wealth	Black-White disparity in farms (1930)	51	0.83 (0.26)	0.97	−1.80 (2.47)	0.966				0.934	0.10 (0.17)	0.020	2.55 (7.54)	0.795				0.632
		Black-White disparity in farms (1940)	51	0.83 (0.25)	0.97	−1.88 (2.82)	0.931				0.866	0.09 (0.16)	0.020	2.63 (8.09)	0.759				0.576
	Wealth	Black-White disparity in value of farms (1930)	51	0.91 (0.15)	0.99	−2.16 (4.04)	0.919				0.844	0.06 (0.10)	0.010	2.16 (4.04)	0.903				0.816
		Black-White disparity in value of farms (1940)	51	0.93 (0.11)	0.98	−1.94 (3.22)	0.923				0.852	0.05 (0.08)	0.010	1.94 (3.22)	0.893				0.797
	Other economic factors	Residential segregation (1930)	51	0.74 (.30)	0.9	−1.143 (0.05)	0.889				0.791	0.12 (0.14)	0.040	1.24 (.354)	0.942				0.887
		Residential segregation (1940)	51	0.79 (0.28)	0.94	−1.47 (0.99)	0.930				0.864	0.11 (0.14)	0.030	1.51 (1.16)	0.932				0.868
		Residential segregation (1950)	51	0.79 (0.26)	0.94	−1.37 (0.71)	0.972				0.944	0.12 (0.16)	0.030	2.21 (6.04)	0.845				0.714
Childhood	Employment	Occupational segregation (1960)	51	0.91 (0.09)	0.94	−1.34 (2.42)		0.937			0.878	0.06 (0.06)	0.040	2.05 (5.97)					
		Occupational segregation (1970)	51	0.90 (0.09)	0.93	−1.03 (0.64)		0.857			0.735	0.06 (0.08)	0.030	3.34 (16.10)					
	Income	Black-White disparity in persons above poverty level (1969)	51	0.85 (0.14)	0.89	−1.72 (3.90)		0.939			0.882	0.07 (0.10)	0.040	4.78 (28.55)					
	Other economic factors	Residential segregation (1970)	51	0.81 (0.23)	0.92	−1.60 (2.04)		0.896			0.802	0.07 (0.11)	0.020	1.75 (1.96)					
Adulthood	Employment	Occupational segregation (1980)	51	0.87 (0.12)	0.89	−1.00 (0.29)			0.816		0.666	0.07 (0.09)	0.050	3.27 (15.38)			0.981		0.963
		Occupational segregation (1990)	51	0.84 (0.17)	0.9	−2.56 (10.14)			0.530		0.281	0.08 (0.09)	0.050	2.80 (11.76)			0.982		0.964
		Occupational segregation (2000)	51	0.83 (0.16)	0.88	−1.30 (1.74)			0.772		0.597	0.09 (0.09)	0.060	1.70 (3.18)			0.847		0.717
	Income	Black-White disparity in persons above poverty level (1979)	51	0.70 (0.15)	0.73	−0.95 (0.27)			0.955		0.912	0.07 (0.09)	0.050	3.29 (15.65)			0.978		0.956
		Black-White disparity in persons above poverty level (1989)	51	0.68 (0.15)	0.71	−0.87 (0.17)			0.950		0.903	0.07 (0.09)	0.050	3.05 (13.70)			0.975		0.950
		Black-White disparity in persons above poverty level (1999)	52	0.64 (0.18)	0.68	−0.73 (−0.11)			0.945		0.892	0.08 (0.08)	0.050	2.07 (6.06)			0.973		0.946
	Wealth	Black-White disparity in aggregate value for owner-occupied units (1990)	51	0.84 (0.19)	0.95	−1.46 (1.06)			0.976		0.952	0.06 (0.12)	0.020	3.18 (12.67)			0.987		0.973
		Black-White disparity in owner-occupied units with 1 or more vehicles (1990)	51	0.82 (0.19)	0.92	−1.42 (1.09)			0.980		0.961	0.06 (11)	0.020	3.05 (11.57)			0.989		0.979
		Black-White disparity in owner-occupied units (1990)	51	0.87 (0.17)	0.96	−2.16 (4.81)			0.935		0.874	0.04 (0.08)	0.010	2.69 (8.22)			0.979		0.959
		Black-White disparity in owner-occupied units (2000)	51	0.83 (0.19)	0.92	−1.74 (2.93)			0.979		0.959	0.06 (0.10)	0.020	2.81 (9.78)			0.991		0.982
	Other economic factors	Residential segregation (1980)	51	0.8 (0.22)	0.92	−1.36 (0.62)			0.960		0.922	0.08 (13)	0.020	2.64 (8.55)			0.991		0.982
		Residential segregation (1990)	51	0.74 (0.16)	0.78	−0.91 (0.43)			0.735		0.540	0.08 (0.13)	0.030	2.47 (7.12)			0.988		0.977
		Residential segregation (2000)	51	0.75 (0.22)	0.85	−1.15 (0.27)			0.980		0.961	0.08 (0.13)	0.030	2.22 (5.24)			0.981		0.962
Midlife	Income	2008–2010 Black-White disparity in average aggregate income for persons 15 years and over in the past 12 months using 2010 inflation-adjusted dollars (2008–2010)	52	0.89 (0.13)	0.95	−1.59 (2.07)				0.968	0.938	0.07 (0.09)	0.030	1.71 (2.42)				0.987	0.975
	Income	Black-White disparity in persons above poverty level in the past 12 months using 2010 inflation-adjusted dollars (2008–2010)	52	0.74 (0.18)	0.81	−1.80 (3.44)				0.986	0.971	0.06 (0.08)	0.020	2.25 (6.17)				0.983	0.966
	Other economic factors	Residential segregation (2010)	52	0.74 (0.20)	0.82	−1.17 (0.68)				0.834	0.695	0.08 (0.12)	0.030	1.91 (3.19)				0.993	0.985

EFA, exploratory factor analysis.

Subsequently, we ran a new CFA model based on life course periods: Before Birth (1930–1950; *K*=11), Childhood/Adolescence (1960–1970; *K*=4), Adulthood (1980–2000; *K*=13), and Midlife (2010; *K*=3). This yielded better fit than the unidimensional model. We then computed four socioeconomic factor scores based on these periods (Table 3). We merged Before Birth factor score with HRS participant state at birth, Childhood/Adolescence with state at age 10, and Adulthood and Midlife with state at interview. Among the sample, the mean Before Birth factor score was −0.618 (SD=1.35), Childhood/Adolescence −0.664 (SD=0.81), Adulthood −0.505 (SD=0.88), and Midlife −0.494 (SD=0.87).

**Table 3.** Descriptive statistics for structural socioeconomic racism and resilience

Life course periods (factors)	***N***	Structural socioeconomic racism							Structural socioeconomic resilience						
		Mean (SD)	Median	Minimum	Maximum	Skewness (kurtosis)	Three lowest scoring states	Three highest scoring states	Mean (SD)	Median	Minimum	Maximum	Skewness (kurtosis)	Three lowest scoring states	Three highest scoring states
Before birth, 1930–1950	51	0.00 (1.05)	0.47	−3.05	1.32	−1.35 (1.08)	Alabama, Mississippi, South Carolina	Massachusetts, New York, Colorado	0.00 (1.01)	0.51	−2.98	0.91	−1.58 (1.60)	Maine, Vermont, New Hampshire	Mississippi, South Carolina, Georgia
Childhood/adolescence, 1960–1970	51	0.00 (.976)	0.22	−3.55	1.01	−1.36 (2.14)	Louisiana, Mississippi, District of Columbia,	New Hampshire, Maine, Vermont							
Adulthood, 1980–2000	51	0.00 (1.00)	0.49	−2.79	1.05	−1.19 (0.444)	Hawaii, Mississippi, District of Columbia	Maine, Vermont, New Hampshire	0.00 (1.01)	−0.42	−0.83	4.9	2.80 (10.37)	Massachusetts, Vermont, Colorado	South Carolina, Mississippi, District of Columbia
Midlife, 2010	52	0.00 (1.00)	0.41	−3.72	0.88	−1.19 (0.444)	Mississippi, South Carolina, District of Columbia	New Hampshire, North Dakota, Wyoming	0.00 (1.00)	−0.44	−0.74	3.71	1.91 (3.44)	Indiana, Nevada, Washington	District of Columbia, Mississippi, South Carolina

### Measure of life course structural socioeconomic resilience

The CFA for the multidimensional model could not converge to produce model fit and the life course model yielded better fit than the unidimensional. Similarly, a three-factor EFA along life course periods was observed (Table 2). We computed factor scores for all life course periods except Childhood/Adolescence, which had communalities that exceeded one during factor rotation. We merged life course factor scores with HRS participant data as with structural socioeconomic racism. Sample mean Before Birth factor score was 0.738 (SD=1.36), Adulthood 0.454 (SD=0.83), and Midlife 0.524 (SD=0.95).

### Life course structural socioeconomic racism and cognitive change over time

In singular life course models, there were significant associations between greater structural socioeconomic racism Before Birth (β=−0.051, 95% confidence interval [CI] −0.094 to −0.009) and during Childhood/Adolescence (β=−0.102, 95% CI −0.176 to −0.029) with cognitive change over time suggesting that as structural socioeconomic racism increased between 1930 and 1970, adults racialized as Black experienced more rapid cognitive decline over 10 years in midlife (Table 4). Although structural socioeconomic racism in Adulthood and Midlife were statistically insignificant, the directionality of the beta coefficients were consistent with the hypothesis.

**Table 4.** Linear mixed effects model of cognitive decline with life course structural socioeconomic racism and resilience

	Structural socioeconomic racism, β (95% CI)	Structural socioeconomic resilience, β (95% CI)
Model per life course period		
Before	−0.051 (−0.094 to −0.009)^*^	0.052 (0.010 to 0.094)^*^
Childhood/adolescence	−0.102 (−0.176 to −0.029)^**^	Did not converge
Adulthood	−0.051 (−0.109 to 0.006)	0.045 (−.017 to 0.106)
Midlife	−0.051 (−0.109 to 0.008)	0.045 (−0.009 to 0.098)
Full model (all life course periods)		
Before Birth	−0.016 (−0.076 to 0.043)	0.043 (−0.007 to 0.092)
Childhood/adolescence	−0.084 (−0.193 to 0.025)	*—*
Adulthood	−0.021 (−0.292 to 0.251)	−0.103 (−0.372 to 0.167)
Midlife	0.022 (−0.256 to 0.300)	0.109 (−.130 to 0.348)
Cisgender category (referent Cisgender male)		
Cisgender female	0.894 (0.499 to 1.288)^**^	0.858 (0.474 to 1.243)^**^
Degree (referent No degree)		
High school degree/GED	2.625 (2.080 to 3.171)^**^	2.639 (2.118 to 3.161)^**^
Some college	4.235 (3.622 to 4.849)^**^	4.291 (3.699 to 4.884)
Age at baseline	−0.108 (−0.212 to −0.003)	−0.083 (−0.184 to 0.019)
Living arrangement (referent married or partnered, living with partner)		
Married or partnered, not living with partner	−0.660 (−1.377 to 0.056)	−0.690 (−1.376 to −0.004)^*^
Not married or partnered, living with unrelated adult	−0.570 (−2.548 to 1.408)	−0.920 (−2.705 to 0.866)
Not married or partnered, living with relative or unrelated minor child	−0.293 (−0.812 to 0.226)	−0.347 (−0.849 to 0.155)
Not married or partnered, living alone	−0.490 (−0.985 to 0.005)	−0.607 (−1.086 to −0.127)^*^

We do not report covariate beta values and CIs for each of the four singular life course period models. We only report the beta coefficients and CIs for the full model with all life course periods. Adjusted models controlled for cisgender category, highest degree obtained, living arrangement and age at baseline.

^**^ *p*<0.01, ^*^p<0.05.

CI, confidence interval; GED, General Education Development.

In the full model, all life course periods were not statistically significant, although directionality for all but Midlife were consistent with the hypothesis. Being cisgender female (β=0.894, 95% CI 0.499 to 1.29; compared to cisgender male) and having a high school diploma (β=2.63, 95% CI 2.08 to 3.17) or some college (β=4.24 95% CI 3.62 to 4.85; compared to no degree)—were all statistically significant covariates in the full model.

### Life course structural socioeconomic resilience and cognitive change over time

Only structural socioeconomic resilience Before Birth was significantly associated with lower cognitive decline at midlife (β=0.052, 95% CI 0.010 to 0.094; Table 4). Similarly, with structural socioeconomic racism, all life course periods were not statistically significant in a full model, but directionality was as hypothesized for all periods except Adulthood. Again, being cisgender female (β=0.858, 95% CI 0.474 to 1.24) and having a high school diploma (β=2.64, 95% CI 2.12 to 3.16) were statistically significant covariates, in addition to living arrangements where married/partnered participants not living with partner (β=−690, 95% CI −1.38 to −0.004) or not married/partnered and living alone (β=−.607, 95% CI −1.09 to −0.127) compared to being married/partnered and living with partner.

## Discussion

Mid-Baby Boomers are a resilient generation that observed a changing world during critical periods of their development. The timing of their childhoods and entry into the workforce presents an ideal opportunity to explore the role of structural racism across the life course, culminating in adverse health in mid-to-late life. In alignment with our hypotheses, these findings suggest that life-course exposure to higher levels of structural socioeconomic racism is correlated with more rapid cognitive decline with aging in midlife. This association is consistent with the lived experiences of persons racialized as Black who have endured structural racism over their life course.^20,25,68–70^ Significant associations between structural socioeconomic racism before birth and during childhood (1930–1970) with rapid cognitive decline highlights the historic impact of structural racism on the health experienced in the present.

Modern-day older adults were born into a Jim Crow segregated society where the socioeconomic resources afforded to persons racialized as White were not equally available to persons racialized as non-White. Although these findings do not make clear whether intergenerational stressors may have been transmitted intrauterine or through epigenetics, it is just as likely that throughout American history, structurally racist policies and practices have persisted over time harming future generations.^60,71^ As research on cognitive aging and dementia shifts focus to early life exposures,^72–84^ there is a grave need to examine multiple life course periods, including before birth to fully understand risk.^85,86^

Building on literature that interrogates the individualization of resilience, we explored structural resilience as an exposure.^30,87^ These findings observed that higher levels of structural socioeconomic resilience is protective against cognitive decline in midlife. Though we believe structural socioeconomic resilience is a form of collective high-effort coping with structural racism that might explain cognitive reserve in midlife for some individuals, we would expect this protective effect to wane over time resulting in more rapid cognitive decline among those with higher levels of socioeconomic racism.^29,88^ Further research is needed to explore such trends. Such research should also include a thorough analysis of intersectional systems of oppression such as structural genderism. These results suggest that cisgender men had worse cognitive outcomes than their cisgender female counterparts.

A comprehensive analysis, that does not inappropriately use being cisgender category as a predictor variable, would allow for greater examination of intersectionality with older cisgender men racialized as Black.

The current study complicates previous approaches that have relied on domain-based and unidimensional models of structural racism, and highlights an important limitation of count-based approaches to quantify structural racism.^89^ The states ranked lowest on structural socioeconomic racism using the ICE approach are in the U.S. South, a region defined by its history of structural racism. Where people racialized as Black live in America is an artifact of structural racism. Because ICE is based on differences in the numbers of persons racialized as White and non-White in a region based on a characteristic (e.g., poverty), differences will tend to be smaller in places with relatively larger numbers of persons racialized as non-White even if the rates of those characteristics are highly disparate between racialized groups. Therefore, count-based approaches, at the county or state level, may not capture the downstream effects of life course structural racism.

Although deserving additional computational scrutiny, the proportion Black approach yielded the finding that greater structural socioeconomic resilience is protective against rapid decline. We expected that experiences of socioeconomic racism in adulthood and midlife would diminish early socioeconomic advantages, although this study's findings did not support this for midlife.^90,91^ These findings illustrate that the health returns to this sample for structural socioeconomic investments before birth are the strongest.

Education, as a structural factor, was not included in these analyses due to discord between how dementia conceptualizes education (e.g., contributing to cognitive reserve) and how structural racism utilizes education (e.g., rewriting history, policing racialized and minoritized children, producing a Black-White academic achievement gap, exacerbating differential access to occupation and livable wages).^32,36,92^ Future research is needed to explore the nuance of these relationships and pathways for a more complete socioeconomic model.

### Limitations

First, the HRS retrospectively collected data from states where participants lived during childhood that are subjective to the recall of participants. Second, we did not have access to state residence information during adulthood and assumed that adulthood and midlife locations were the same. Thus, the score in adulthood may not capture this generation's absolute experience with structural racism. Third, various measurement issues in census and cognitive data could have induced bias in our findings. Small sample size (*N*=52) likely contributed to poor model fit in the CFAs. However, our sample size is fixed by the geopolitical units of the United States. Expectations around model fit must be reconsidered to capture the historic role of states with policies that drive life course structural racism. Fourth, the restriction of statistical variance that undergirds most models is problematic for the study of racism.

Conceptually, structural racism may vary in its application and impact, but it does not vary in its measured magnitude in a way that is well-suited for statistical analysis. Thus, it was unsurprising that neither ICE nor proportion Black yielded a diverse range of values. Hence, there were measurement challenges with examining differences across geographic areas in a society without structural racism since inception. Future research must grapple with the issue of variance in inferential statistics, as well as survivor bias, toward studying the impact of structural racism on racialized and minoritized populations. Although we did not adjust for attrition due to transition or discontinuance with the study, doing so may provide salient information in longitudinal analyses. Although care must be taken with this approach as structural racism often interferes with the morbidity and mortality of participants racialized as Black and cannot always be controlled for.

## Conclusion

The socioeconomic context in the United States shapes the distribution of education access, occupations and income, and other dementia risk-reducing resources for people across the life course.^68,92,93^ Structural racism via socioeconomic opportunities shaped governmental action and inaction at local, state, and federal levels in ways that dictate how people racialized as Black gain access to resources to increase quality of life.^11,94–96^ Generations of *de jure* residential segregation have taken their toll on holistic health of people racialized as Black,^38,97,98^ setting a deleterious trajectory of racialized life experiences that leave an imprint on cognitive function later in life.^92,99^ Addressing the disproportionate socioeconomic opportunities, at minimum, will have reverberating effects throughout the life course. The most impactful change may come from a close examination of past policies and practices that maintain structural socioeconomic racism that can be reversed to prevent their predictive impact on the cognitive health of future generations.

## Data Availability

These data are publicly available at IPUMS and the Health and Retirement Study.

## Notes

**Cite this article as:** Adkins-Jackson PB, Kim B, Higgins Tejera C, Ford TN, Gobaud AN, Sherman-Wilkins KJ, Turney IC, Avila-Rieger JF, Sims KD, Okoye SM, Belsky DW, Hill-Jarrett TG, Samuel L, Solomon G, Cleeve JH, Gee G, Thorpe Jr., RJ, Crews DC, Hardeman RR, Bailey ZD, Szanton SL, Manly JJ (2024) “Hang ups, let downs, bad breaks, setbacks”: impact of structural socioeconomic racism and resilience on cognitive change over time for persons racialized as Black, *Health Equity* 8:1, 254–268, DOI: 10.1089/heq.2023.0151.

## Abbreviations Used

CFA: confirmatory factor analyses
CI: confidence interval
EFA: exploratory factor analysis
GED: General Education Development
HRS: Health and Retirement Study
ICE: index of concentration at the extremes
KMO: Kaiser-Meyer-Olkin
SD: standard deviation
TICS: Telephone Interview for Cognitive Status
